# A novel method for automatic classification of Parkinson gait severity using front-view video analysis

**DOI:** 10.3233/THC-191960

**Published:** 2021-07-09

**Authors:** Taha Khan, Ali Zeeshan, Mark Dougherty

**Affiliations:** a Centre for Artificial Intelligence, School of Information Technology, Halmstad University, Halmstad, Sweden; b Department of Computer Science, FAST-National University, Karachi, Pakistan

**Keywords:** Parkinson’s disease, gait impairment, computervision, motion analysis

## Abstract

**BACKGROUND:**

Gait impairment is an essential symptom of Parkinson’s disease (PD).

**OBJECTIVE:**

This paper introduces a novel computer-vision framework for automatic classification of the severity of gait impairment using front-view motion analysis.

**METHODS:**

Four hundred and fifty-six videos were recorded from 19 PD patients using an RGB camera during clinical gait assessment. Gait performance in each video was rated by a neurologist using the unified Parkinson’s disease rating scale for gait examination (UPDRS-gait). The proposed algorithm detects and tracks the silhouette of the test subject in the video to generate a height signal. Gait features were extracted from the height signal. Feature analysis was performed using the Kruskal-Wallis rank test. A support vector machine was trained using the features to classify the severity levels according to UPDRS-gait in 10-fold cross-validation.

**RESULTS:**

Features significantly (p< 0.05) differentiated between median-ranks of UPDRS-gait levels. The SVM classified the levels with a promising area under the ROC of 80.88%.

**CONCLUSION:**

Findings support the feasibility of this model for Parkinson’s gait assessment in the home environment.

## 1. Introduction

Parkinson’s disease (PD) deteriorates motor functions and develops gait symptoms over time. These symptoms include short-shuffling steps, postural instability, slow walking, etc. [1]. Parkinsonian gait is clinically examined using the unified Parkinson’s disease rating scale part-III item-29 (UPDRS-gait) [2]. This examination requires a patient to walk back and forth on a 10 meters gait platform. A doctor rates the walk on a scale of ‘0’ and ‘4’ using UPDRS-gait. ‘0’ represents a healthy walk ‘1’ represents a slow walk with shuffling steps ‘2’ represents a walk with shortshuffling steps and festination. ‘3’ represents a severe gait disturbance that requires assistance for walking ‘4’ indicates total disability to walk even with assistance.

Some limitations of examining PD include the consumption of extensive time and resources of healthcare systems [3], the physical ability of patients to visit clinics for regular assessment, and subjective evaluation of symptoms by a doctor that is prone to human error. A solution is to employ vision-based telemonitoring tools to enable continuous monitoring of patients in their home environment.

**Figure 1.**
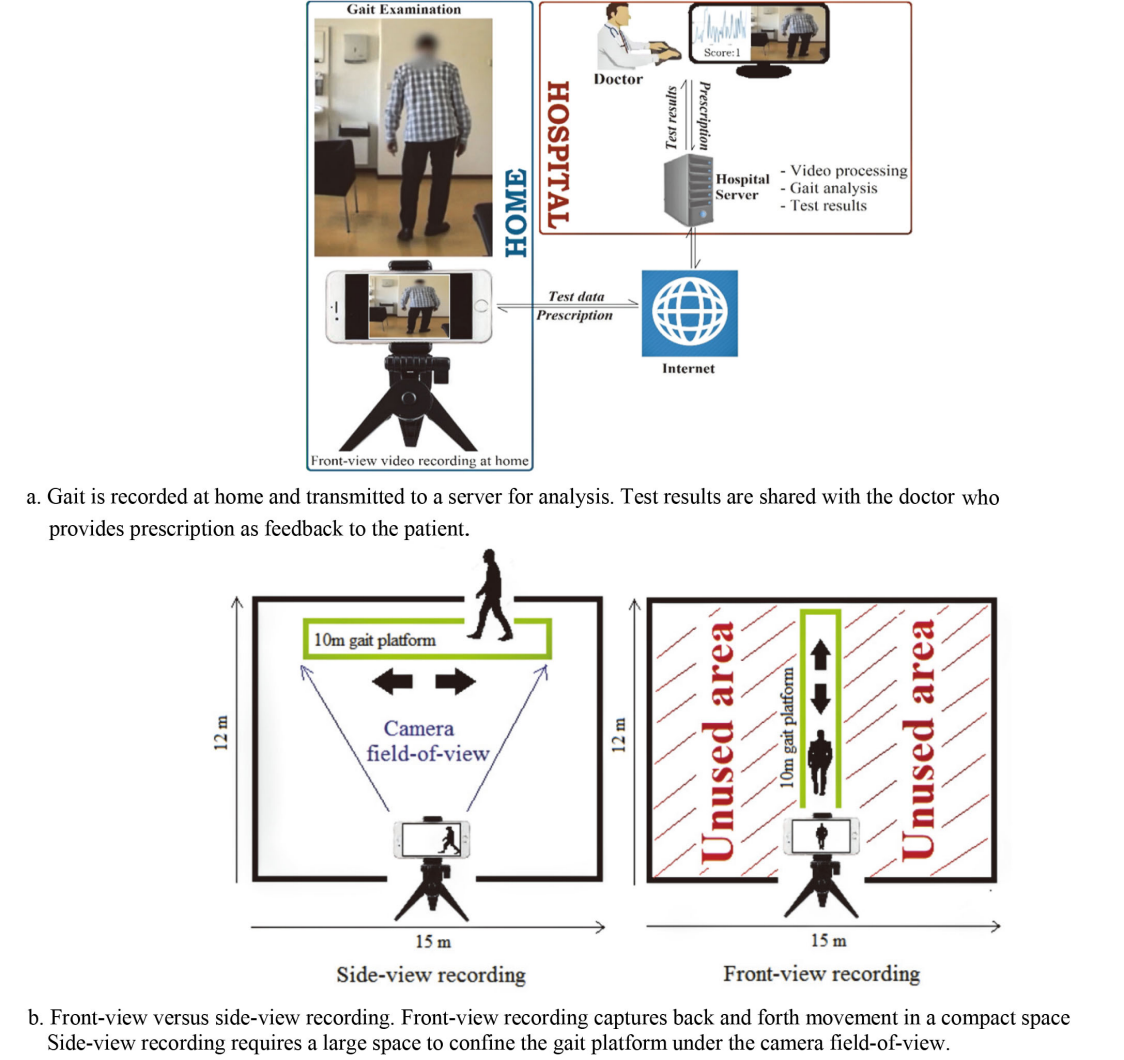
At-home gait assessment based on front-view video analysis.

State of the art vision-based methods of gait analysis used Kinect sensors [4]. However, Kinect sensors are not commonplace as compared to the RGB cameras in smartphones, laptops, and tablets. Importantly, these devices allow transmitting recordings of gait to a server where videos can be processed, and results presented to a caregiver. Subsequently, the prescription can be transmitted back to the patient’s device (Fig. 1a). This feedback mechanism of computerized gait assessment improves interactivity between patients and caregivers that allows timely treatment of patients at home not possible through conventional manual ways of treating Parkinson’s disease.

A recent study [5] used an RGB camera to record side-view of test subjects to obtain visual separation of legs for estimating steplength. The study reported a strong correlation between fall and steplength. Similarly, another study [6] used an RGB camera and side-view for estimating the silhouette of a walking person and suggested that gait analysis can be performed without a lab, or physical attachment of sensors or markers to the patients.

However, a disadvantage of side-view assessment is that a large room is needed for recording (Fig. 1b). Alternatively, recording from front confines the back and forth movement of the subject within the camera field-of-view, which allows recording gait in compact spaces such as corridors. This is important because studies suggest that freezing and falling is less likely to occur in corridor walks since corridors provide visual cues to the patients that assist them in planning their movement [7].

We propose a machine learning model for estimating Parkinsonian gait symptoms using front-view video analysis. The method follows the UPDRS protocols and allows using a compact space. The algorithm uses the varying height of the subject in a sequence of video frames to extract features representing gait symptoms. A support vector machine (SVM) was trained using these features to score the severity of gait impairment based on the UPDRS-gait.

## 2. Method

### 2.1 Data acquisition

Data were acquired between 2002 and 2003 at five clinics in Sweden in a study entitled ‘Duodopa Infusion: Randomized Efficacy and Quality-of-life Trial’ [8]. In the study, gait examinations of 24 patients (19 males and 5 females) were videotaped. The patients were aged between 50 and 75 and had a mean total-UPDRS score of 50.45 on a scale between ‘0’ (healthy) and ‘108’ (total disability).

Gait examination was conducted in a 10 meters long corridor. Patients were seated on one end of the corridor and a camera is pivoted at the other end. Patients were asked to rise from the chair, walk straight to the camera, turn, and walk back to the chair. The gait was recorded, and the video was transmitted to a server accessed by a neurologist. The neurologist watched the video and rated the walking performance based on the UPDRS-gait.

Each patient was examined and videotaped 17 times throughout the day with a rest of half-an-hour before each examination. Videos of patients with a total disability to walk (rated ‘4’) and those who required assistance (rated ‘3’) were not used for the analysis due to the interference of nursing assistants in the videos. Also, some patients dropped out of the study. The videos were recorded at 25 frames per second and a resolution of 352 × 288 pixels. Written informed consent was obtained from all patients. 

Since multiple videos were recorded of an individual, to avoid subjective bias in model development and to balance sample distribution, 456 videos with reasonable quality (no blur/shadows/highlights/occlusion) were randomly selected from the database such that classes ‘0’, ‘1’ and ‘2’ consisted of 152 samples each. The videos were used for method validation and analysis.

### 2.2 Method description

**Figure 2.**
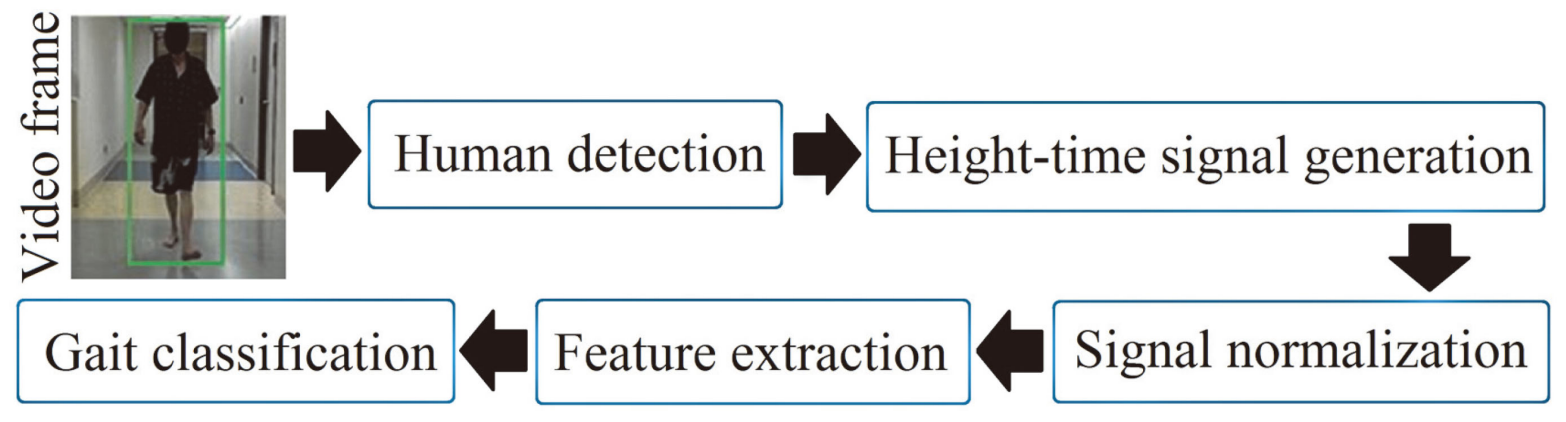
Block diagram of the gait algorithm.

The block diagram of the algorithm is shown (Fig. 2). In the first step, the test subject was identified in the video using a human detector based on the histogram-of-oriented gradients (HOG) [9]. HOG returns a bounding-box that confines the height and width of the subject in a video frame. In the second step, a height signal was produced by using the varying height of the boundingbox in a sequence of video frames. The signal was height-adjusted and normalized. Features were extracted from the height signal for training an SVM to score UPDRS-gait. The steps are described further.

**Figure 3.**
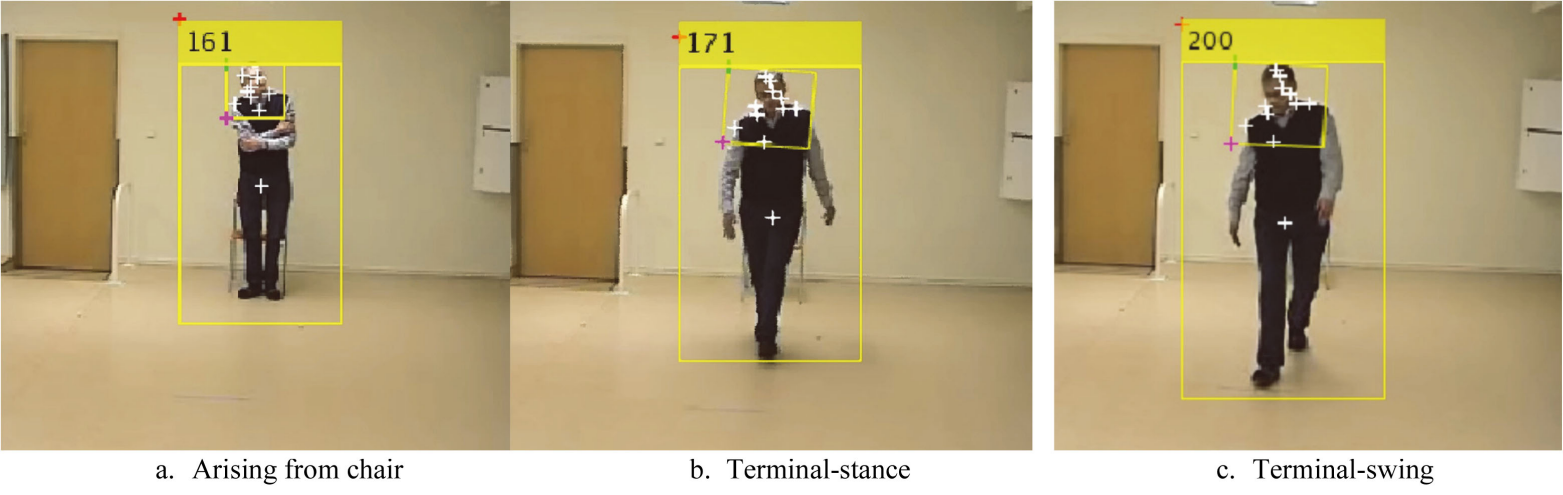
Human detection in an image sequence. $S_{h\,i}$ in pixels is shown in the top-left corner of the bounding box that tends to increase with each forward step towards the camera.

#### 2.2.1 Human detection

Human detection using HOG [9] is based on the idea that the appearance of a local object in an image can be characterized by the distribution of gradients of pixel intensities. A significant intensity difference across pixels indicates an edge. The algorithm operates by dividing an image into connected regions called cells. A local 1-D histogram of pixel intensities in that cell is computed. The histogram is contrast-normalized using the Gaussian weight of pixel intensities across larger regions of the image referred to as blocks (Eq. (1)).

(1) $$G\,\left(x\right)=e^{\left(-\frac{{\left(x-\mu \right)}^{2}}{2\,\sigma^{2}}\right)}$$

Where x is pixel intensity, μ is the mean pixel intensity in a block and σ is the standard deviation of pixel intensities in that block. These normalized histograms of cells of a block are termed as HOG descriptors that are collectively used as features for training an SVM to detect human presence in an image. The method was previously tested on the MIT pedestrian database [10] consisting of 509 training and 200 test images of walking pedestrians, as well as the INRIA database [9] consisting of 1805 test images of human poses. The method successfully detected human in both databases with zero miss rate. In our study, the cell size was 6 × 6 pixels, and the block size was 3 × 3 cells. The method detected walking subjects in our video recordings with 100% accuracy.

#### 2.2.2 Height signal 

The HOG algorithm returns a bounding-box with height $S_{h}$ and width $S_{w}$ of a human silhouette in an image. $S_{h}$ increases when the subject walks closer to the camera and decreases when he walks away (Fig. 3). $S_{h}$ remains constant when left and right legs are adjacently positioned during mid-swing and mid-stance phases and increases when both legs are positioned apart during terminal-swing and terminal-stance (Fig. 5b). $S_{h}$ generates a height signal $S_{h\,i}$ for a video-frame sequence i= 1 to n total frames.

#### 2.2.3 Signal pre-processing

For accurate estimation of gait symptoms, the method must be robust to varying heights of people since gait attributes are affected by height. For instance, a tall person’s stride is generally longer than a short person’s stride. To account for the height variation, $S_{h\,i}$ was scaled using a human model [11]. According to this model, face height $f_{h}$ is proportional to total height $S_{h}$. A face detector [12] was used to compute $f_{h}$. $S_{h\,i}$ was height-adjusted by dividing $S_{h\,i}$ by $f_{h}$ in each video frame to produce $S_{h\,i}^{\prime }$. Also, recordings using a camera placed closer to the gait platform produces higher $S_{h}$ than if the camera is placed farther. To accommodate varying camera positions, $S_{h\,i}^{\prime }$ was normalized between 0 and 1 using Eq. (2).

(2) $$\,S_{h\,i}=\frac{S_{h\,i}^{\prime }-\text{min}\,\left(S_{h\,i}^{\prime }\right)}{\text{max}\,\left(S_{h\,i}^{\prime }\right)-\text{min}\,\left(S_{h\,i}^{\prime }\right)}\;\text{ for }\,i=1\,\ldots \,n$$

**Figure 4.**
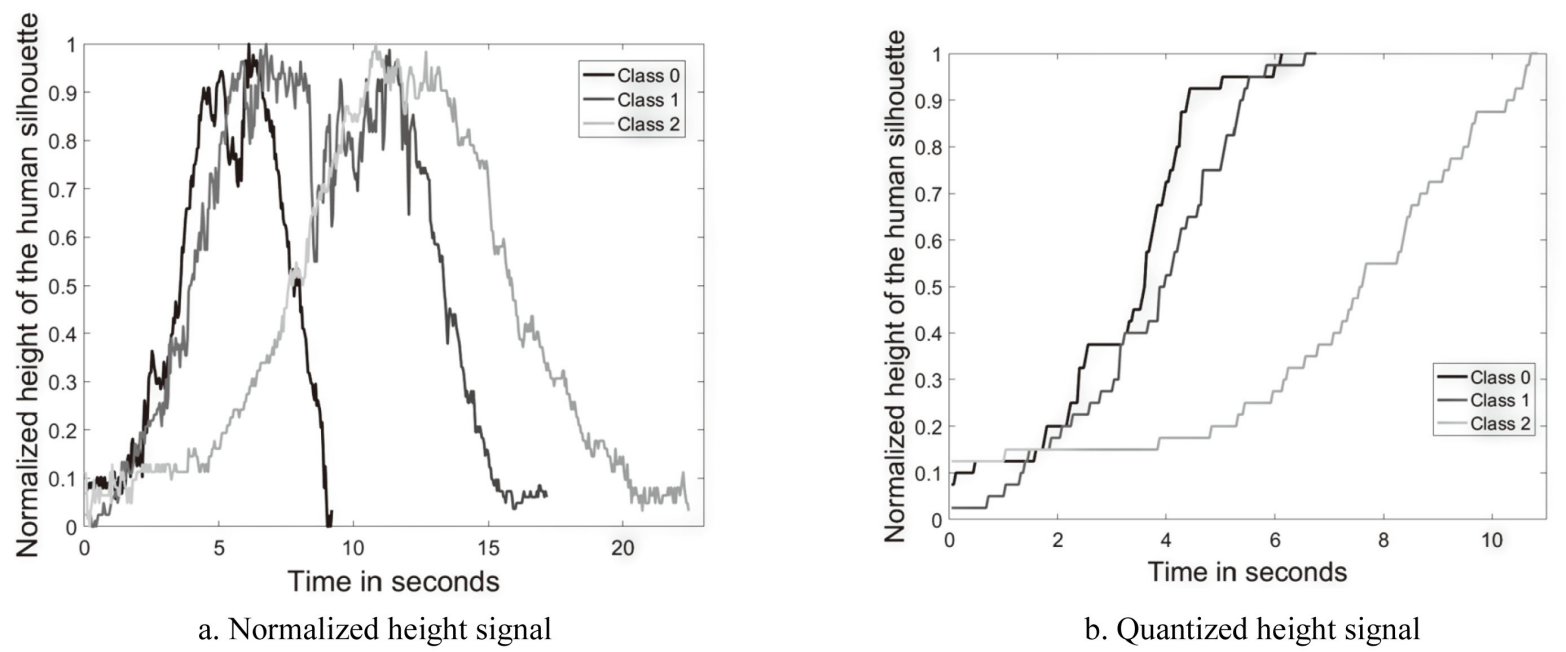
The height signal $\,S_{h\,i}$ is shown for representative samples rated ‘0’ (healthy), ‘1’ (mildly-impaired), and ‘2’ (moderately-impaired). 4a shows normalized $\,S_{h\,i}$ of a complete walk, i.e., walking forward from the initial position, turning and walking back to the initial position. 4b shows quantized $\,S_{h\,i}$ of a forward walk.

The normalized signal $\,S_{h\,i}$ of representative videos rated ‘0’ (healthy), ‘1’ (mildly-impaired), and ‘2’ (moderately-impaired) are shown (Fig. 4a). It was observed that the completion time of gait, i.e., time taken in walking forward from the initial position, turning and walking back to the initial position, was lowest for healthy, higher for mildly-impaired, and highest for moderately-impaired gait. Small-shuffling steps in impaired gait signals were noticed, i.e., signals showed smaller amplitude changes compared to healthy gait. Turning time was lowest in healthy gait. Importantly, the healthy gait signal showed quick and smooth progress compared to impaired gait signals

To remove signal aberrations, $\,S_{h\,i}$ was smoothed using a moving-average filter and quantized using the Lloyd algorithm [13]. The algorithm approximates a continuous set of values within a signal partition and maps them to one discrete weighted-average-centroid of points in that partition. A partition size of five points was selected for quantization. Gait events were approximated using increasing values of the quantized signal $\,S_{h\,i}$ representing a forward walk. To do this, $\,S_{h\,i}$ was split between the forward and backward walks by using the maximum height value that represents the position where the subject is closest to the camera. The forward walk $\,S_{h\,i}$ in representative videos are shown (Fig. 4b).

#### 2.2.4 Feature extraction

Stride has two phases, swing and stance. As discussed above, $S_{h}$ remains constant during mid-swing and mid-stance and increases during terminal-swing and terminal-stance. Strides were approximated using $\,S_{h\,i}$ to compute features representing level-1 symptoms of slow walking and short-shuffling steps, and level-2 symptom of gait-festination. First, stance time (S⁢T) was computed using Eq. (3).

(3) $$S\,T\,\left(i\right)=T_{s\,\left(i+1\right)}-T_{s\,\left(i\right)}$$

**Figure 5.**
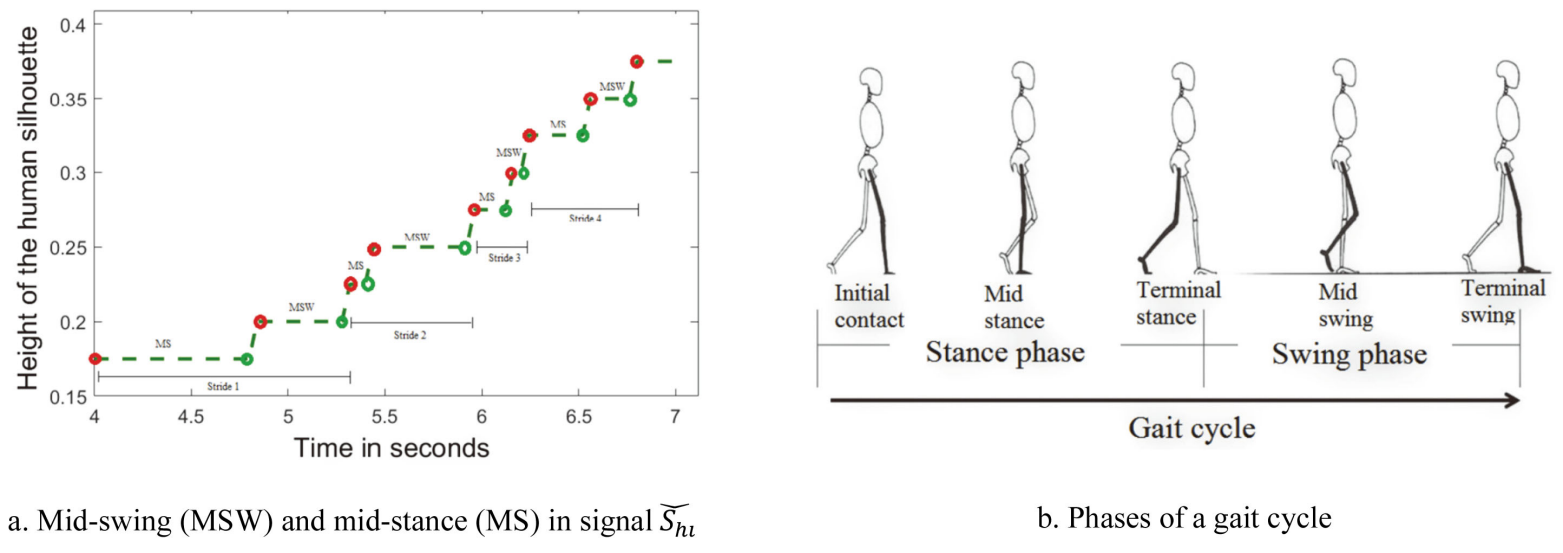
Representation of gait events in the quantized height signal.

Where $T_{s\,\left(i\right)}$ is the timestamp in $\,S_{h\,i}$ that represents the initial contact of the front foot with the ground. Timestamp $T_{s\,\left(i+1\right)}$ is the point of amplitude increase in $\,S_{h\,i}$ that represents the terminal-stance (Fig. 5b). Now, swing time (S⁢W) was computed using Eq. (4).

(4) $$S\,W\,\left(i\right)=T_{s\,\left(i+2\right)}-T_{s\,\left(i+1\right)}$$

Where $T_{s\,\left(i+1\right)}$ is the timestamp in $\,S_{h\,i}$ representing the initial swing. Timestamp $T_{s\,\left(i+2\right)}$ is the point of amplitude increase in $\,S_{h\,i}$ that represents the terminal-swing (Fig. 5b). Finally, stride time ($S_{T}$) was computed using Eq. (5).

(5) $$S_{T}\,\left(j\right)=S\,T\,\left(i\right)+S\,W\,\left(i\right)\;\text{ for }\,i=1,3,5\,\ldots \,n-2,\text{ and }\,j=1\,\ldots \,N\,\text{ total strides}$$

To estimate short steps indicating level-1 impairment, average stride time $S_{\text{𝑎𝑣𝑔}}$ was computed using Eq. (6). Low $S_{\text{𝑎𝑣𝑔}}$ indicates an overall short step-length.

(6) $$S_{\text{𝑎𝑣𝑔}}=\frac{1}{N}\,\sum_{j=1}^{N}S_{T}\,\left(j\right)$$

Detrended fluctuation analysis (DFA) and entropy E were used to estimate step shuffling. DFA determines signal self-affinity using long-range correlations. This was done by integrating signal $\,S_{h\,i}$ using Eq. (7).

(7) $$y\,\left(k\right)=\sum_{i=1}^{k}\left[\,S_{h\,i}-S_{\text{𝑎𝑣𝑔}}\right]\;\text{ for }\,k=1\,\ldots \,N$$

Where y⁢(k) is the integrated signal. y⁢(k) was divided into boxes of equal length l. We kept l= 5. For each box, a least-square fit and y-coordinates of the fitted-line $y_{n}\,\left(k\right)$ were computed. Fluctuation F⁢(l) was measured for total boxes L using Eq. (8).

(8) $$F\,\left(l\right)=\sqrt{\frac{\sum_{k=1}^{L}{\left(y_{k}-y_{n}\,\left(k\right)\right)}^{2}}{L}}\;\text{ for }\,l=l,2\,l,3\,l\,\ldots \,L/l$$

Self-similarity α was computed as the slope of the log-log plot between F⁢(l) and l. α equals 1 if the boxes are similar, or lesser or greater than 1 otherwise [14].

Entropy in $\,S_{h\,i}$ was computed using Eq. (9).

(9) $$E=-\sum \,S_{h\,i}\times \log_{2}\left(\,S_{h\,i}\right)$$

Spectral centroid variability in $\,S_{h\,i}$ was computed to estimate gait-festination indicating level-2 impairment. Quick abrupt short steps accompanied by imbalance characterize gait-festination. This means that a level-2 signal should have higher randomness as well as sharp shifts in signal values, meaning weak frequency centroids across the signal compared to healthy gait. Spectral centroids were computed for a total of N boxes of box size n= 5 using Eq. (10).

(10) $$C_{i}=\frac{\sum f_{i}\,x_{i}}{\sum x_{i}}\;\text{ for }\,i=1\,\ldots \,N$$

Where $f_{i}$ is the frequency in Hertz and $x_{i}$ are spectral values in the $i^{\text{th}}$ box. Centroid variability for estimating abrupt short steps was computed as the mean difference between consecutive centroids given as

(11) $$A_{\Delta \,C}=\frac{\sum_{i=2}^{N}\left(C_{i}-C_{i-1}\right)}{N}$$

Slow walking was estimated by computing time T between the valley and peak of the signal $\,S_{h\,i}$. A total of five features 1) $S_{\text{𝑎𝑣𝑔}}$, 2) α, 3) E, 4) $A_{\Delta \,C}$ and 5) T representing UPDRS-gait symptoms were used for training an SVM to classify UPDRS-gait.

#### 2.2.5 Feature analysis

A non-parametric one-way analysis of variance of features across severity levels was performed using the Kruskal-Wallis test [15]. For each feature, the test ranked feature values from smallest to largest. Level mean-ranks were compared to test the null-hypothesis that independent samples belong to continuous distributions that are indistinguishable. Statistical significance (p< 0.05; 95%CI) was computed to identify if features truly represent UPDRS-gait symptoms and discriminate severity levels based on mean-ranks. Results are given in Section 3.

#### 2.2.6 Classification

SVM was chosen for its ability to find optimal margins between class boundaries over a high-dimensional feature space [16]. SVM uses a kernel function for mapping features to a higher dimension by using images of the inner product between pairs of features, which is computationally inexpensive compared to computing actual feature coordinates in a high-dimensional space. We used a PUK kernel $k\,\left(V_{i}\,V_{j}\right)$ [17] that is a modified form of the Pearson VII Gaussian function given in Eq. (12).

(12) $$k\,\left(V_{i},V_{j}\right)=\frac{1}{{\left[1+{\left(\left(2\,\sqrt{{\left|V_{i}-V_{j}\right|}^{2}}\,\sqrt{2^{1/\omega }-1}\right)/\sigma \right)}^{2}\right]}^{\omega }}$$

Where $V_{i}$ and $V_{j}$ are training feature vectors, σ adjusts the half-width of the peak of the Gaussian curve, and ω controls the tailingfactor of the peak. A feature matrix of 5 features × 456 samples were used to train the SVM to classify UPDRS-gait levels ‘0’, ‘1’, and ‘2’. For this multi-class classification problem, a one-vs-all classification approach was used such that the SVM was trained to discriminate between samples of a class versus samples of the other two classes. Hence three models were developed to classify ‘0’, ‘1’, and ‘2’ separately. The training performance was optimized by tuning σ and ω.

To avoid biased generalization, data were stratified using 10-fold cross-validation i.e., the models were trained and tested in 10 iterations. In each iteration, 90% of randomly selected samples were used for training and 10% were used for testing the model. Samples used for testing once are not repeated for testing in other iterations. Prediction accuracy was computed for each iteration and results are averaged over ten iterations. The overall performance was evaluated using confusion matrices and ROC curves. Results are given in Section 3. 

## 3. Results

**Figure 6.**
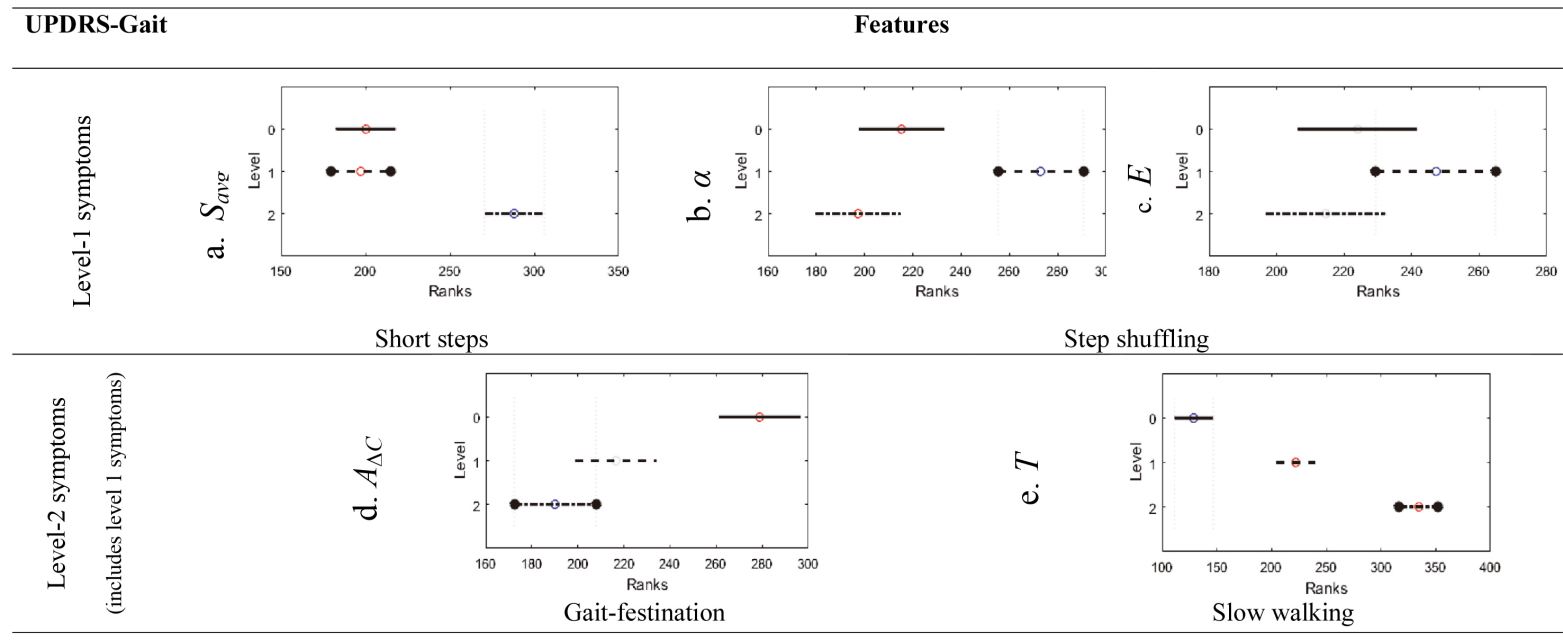
Feature analysis using the Kruskal-Wallis mean-rank test. Bars bordered with circles represent the UPDRS-gait severity level under which a symptom is examined.

A comparison between feature mean-ranks of UPDRS-gait levels is shown (Fig. 6). Feature $S_{\text{𝑎𝑣𝑔}}$ estimates short steps, which is a level-1 symptom. The test confirmed that the mean-ranks of $S_{\text{𝑎𝑣𝑔}}$ was the lowest in level-1 and significantly different (p-value = 6.37 × 10${}^{-11}$) than the mean-ranks of level-2. However, the mean-ranks of levels 0 and 1 were not significantly different.

Features α and E estimate step shuffling which is a level-1 symptom. The level-1 mean-ranks of α was significantly higher (p-value = 1.13 × 10${}^{-6}$) than the mean-ranks of level 0 and 2; however, the mean-ranks of 0 and 2 were not significantly different. Also, feature E mean-ranks was the highest in level-1 although insignificantly (p-value = 0.085).

Feature $A_{\Delta \,C}$ estimates gaitfestination that is a level-2 symptom. Results affirmed that $A_{\Delta \,C}$ mean-ranks in level-2 were significantly (p-value = 1.20 × 10${}^{-8}$) lower than the mean-ranks of levels 0 and 1. Also, feature T discriminated between the mean-ranks of the three levels with statistical significance (p-value = 7.86 × 10${}^{-41}$) suggesting that walking speed reduces with severity of gait impairment.

**Figure 7.**
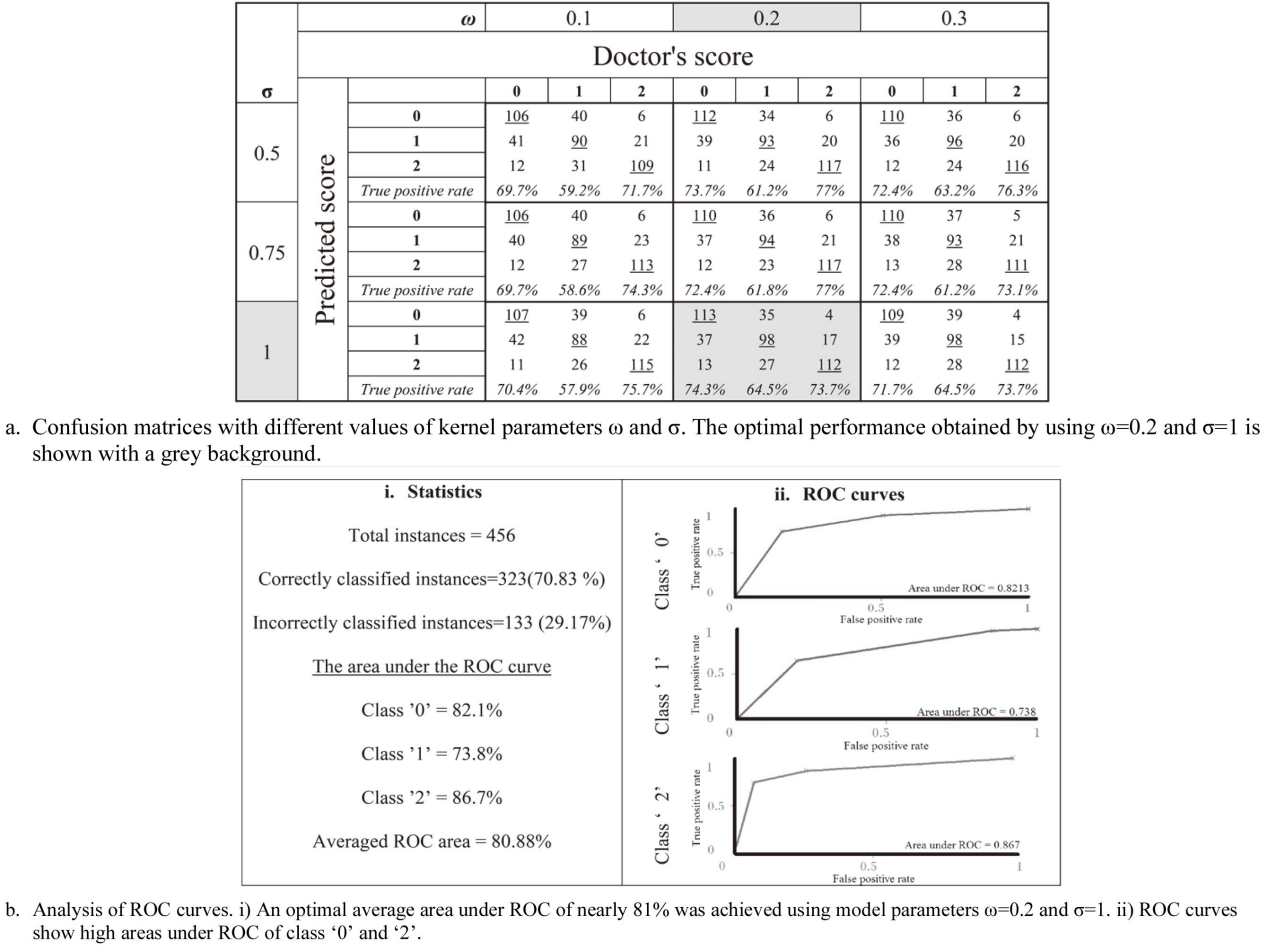
Classification performance of the SVM model.

The SVM model trained using these features and tuned using model parameters (ω= 0.2; σ= 1.0) predicted the UPDRS-gait scores with an averaged accuracy of 70.83% (Fig. 7). Reasonable true-positive rates were produced for classes ‘0’ (74.3%), ‘1’ (64.5%), and ‘2’ (73.7%). The averaged area under the ROC curves of 80.88% was promising. Moreover, the ROC curves of class ‘0’ and ‘1’ were protruded upwards, supporting the model’s ability to classify class ‘0’ and ‘2’ with high accuracy.

## 4. Conclusions

We introduced a new method of Parkinson’s gait assessment using front-view video analysis. The method computes the varying height of the human silhouette in video frames and quantizes the height signal to estimate temporal gait features. Important features significantly (p< 0.05) represented gait symptoms of short-shuffling steps and festination that are clinically observed by a doctor to rate mild, moderate and severe stages of Parkinson’s gait. Moreover, the SVM model correctly predicted the UPDRS-gait scores with a high average area under ROC curves.

Recent work based on Kinect sensors [18] supports that front-view analysis saves space for gait assessment compared to side-view that requires large space. However, Kinect sensors are not commonplace. By contrast, our methodology used an RGB-camera available in devices used in everyday life, facilitating gait assessment in narrow corridors at home with no specialised equipment. Moreover, the algorithm is a low-cost alternative to motion capture systems for PG assessment, such as [19], that requires advanced equipment and a controlled environment.

In conclusion, the proposed SVM model and features accurately characterized the severity of gait impairment according to UPDRS standards without requiring complicated lab settings and the need for physical attachment of body markers and sensors. The excellent accuracy obtained in the classification of UPDRS-gait severity levels and importantly, the significant ability of features to characterize the severity, suggest that the model can be used for clinical evaluation in non-laboratory settings, can support in tracking gait symptoms and help in treatment interventions.

Future work includes optimizing the framework by incorporating biomechanics such as leg joints, angles, hand movements, etc. made possible by recording videos at higher speed and resolution. The study could be expanded to examine gait problems in other neurological disorders such as Huntington’s disease, neuropathy, or rehabilitation after lower limb surgeries. Also, deep learning can be used for model development by recording a larger dataset of gait videos for training the classifier. We plan to integrate the proposed method to a test battery system [20] that allows telemonitoring of activities of daily living of patients to enable an overall PD assessment.
